# Antibiotic prophylaxis habits in oral implant surgery among dentists in Italy: a cross-sectional survey

**DOI:** 10.1186/s12903-019-0943-x

**Published:** 2019-12-02

**Authors:** Fabio Rodríguez Sánchez, Iciar Arteagoitia, Carlos Rodríguez Andrés, Alfonso Caiazzo

**Affiliations:** 1 Department of Epidemiology and Public Health, University of the Basque Country (UPV/EHU), Bilbao, Spain; 2 Department of Oral Health Sciences, Periodontology, Catholic University of Leuven & University Hospitals Leuven, Leuven, Belgium; 3 Department of Stomatology, University of the Basque Country (UPV/EHU), Bilbao, Spain; 4 BioCruces Health Research Institute member, Cruces University Hospital, Barakaldo, Spain; 5 Henry M. Goldman School of Dental Medicine (Boston University), Boston, USA; 6 Private Practice Salerno, Salerno, Italy

**Keywords:** Antibiotic prophylaxis habits, Oral implant surgery, Postoperative infection, Bacterial resistance

## Abstract

**Background:**

The prescription of prophylactic antibiotics in conjunction with oral implant surgery remains inconsistent among different populations of dentists. The main objective of this study was to assess the current antibiotic prescribing habits of dentist in conjunction with oral implant surgery in Italy. The secondary objective was to assess the nature and amount (mg) of antibiotics prescriptions in order to evaluate whether any consensus has been reached and if the current recommendations are complied.

**Methods:**

Observational cross-sectional study based on a web-survey reported according to the STROBE guidelines. A questionnaire was sent via email to each registered member of the Italian Academy of Osseointegration (*n* = 400). The email included a link to the anonym web questionnaire developed on www.encuestafacil.com. It contained close-ended and some open-ended questions concerning demographics, antibiotic type, prescription duration and dosage. Collected data were analyzed using STATA® 14 software.

**Results:**

160 participants responded the survey (response rate = 40%). Approximately 84% routinely prescribed prophylactic antibiotics in conjunction with oral implant surgery, 15.6% prescribed antibiotics in certain situations and only 1 did not prescribe antibiotics at all. Overall, 116 respondents prescribed both pre- and postoperative antibiotics, 29 prescribed antibiotics only preoperatively and 14 prescribed antibiotics exclusively after surgery. Italian dentists prescribed an average amount of 10,331 mg antibiotics before, during or after oral implant surgery. Approximately, only 17% (*n* = 27) of the participants who prescribed antibiotics before oral implant surgery complied with the recommendations proposed by the latest publications (no more than 3 g of preoperative amoxicillin before oral implant surgery).

**Conclusions:**

Dentists in Italy on a large scale prescribe antibiotic prophylaxis in conjunction with oral implant surgery among healthy patients. A high range of prophylactic regimens is prescribed and they are not adhering to the new science-based specifications. Guidelines focused on the indications for prophylactic antibiotics among healthy patients are required to prevent bacterial resistance, side effects and costs caused by overtreatment and the irrational use of antibiotics.

## Background

Oral implant surgery is a routine treatment from which both dentists and patients expect high success rate, but often this is not the case [1, 2]. Bacterial contamination at implant surgery has been related to early implant failures [3]. Therefore, different prophylactic treatments such as the use of perioperative antibiotics have been studied [4].

Nevertheless, the use of prophylactic antibiotics to reduce the incidence of postoperative infections and oral implant failures in healthy patients is still controversial [5, 6]. Several reviews have found no evidence supporting the prophylactic effect of antibiotics on postoperative infections, and they have remained inconclusive on the prevention of oral implant failures [2, 7–12]. Consequently, many professionals disagree on the utility of antibiotics and which is the most suitable regimen to adopt [13–18].

The inadequate use of antibiotics must be seriously taken into consideration as it could cause bacterial resistance and other important adverse effects, such as secondary infections, interactions with other medications, gastro-intestinal discomfort, toxicity and allergic reactions [7, 18]. The consequences are substantially human and economic [19].

Owing to this, the use of antibiotics has been the subject of a special monitoring in the European Union (EU) and the main topic of public awareness campaigns [20]. Italy was the ninth country with more systemic consumption of antimicrobials in the EU community (primary care sector) in 2017 [21]. Moreover, Italy was one of the countries with the highest levels of bacterial resistance in most pathogenic species monitored [22].

There is evidence showing that dental practitioners have over prescribed large numbers of systemic antibiotics and that their number has even increased in the last years [23]. In addition, a recent survey involving more than one thousand Italian dentists found that the use of systemic antibiotics is frequent and excessive [24].

In Italy, dental practitioners whether specialized or not in periodontics or oral surgery routinely perform oral implant surgery. Implant training is part of the basic training as dentists as well as part of the postgraduate in oral surgery and periodontology.

There used to be two scientific bodies related to oral implatology: the Italian Society of Oral Surgery and Implant Dentistry (SICOI) and the Italian Society of Osseointegration (SIO). In 2015, they merged into a new entity called the Italian Academy of Osseointegration (IAO). Unfortunately, there is still no guideline available regrading antibiotic prophylaxis in oral implant surgery in Italy. Nevertheless, new research assessing the effectiveness of prophylactic antibiotics for oral implant surgery among healthy patients has been published. They recommended a single dose (1 g, 2 g or 3 g) of oral Amoxicillin preoperatively [2, 7]. Moreover, several studies assessed the antibiotic prescribing patterns in conjunction with oral implant surgeries in different countries [13–18].

However, in Italy this issue has yet to be addressed. Therefore, it is currently important to evaluate the different regimens adopted among oral health professionals in Italy in comparison to other countries.

The primary aim of this study was to determine whether antibiotic prophylaxis is a common treatment in Italy among dentists in conjunction with oral implant placement in healthy patients. The secondary aim was to assess the nature and amount (mg) of antibiotics prescriptions in order to evaluate whether any consensus has been reached and if the current recommendations supported by last published evidence are complied [2, 7].

## Methods

This observational cross-sectional study is based on a web survey and it is reported according to the Strengthening the Reporting of Observational studies in Epidemiology (STROBE) guidelines [25].

### Study design

The questionnaire developed by Deeb et al. (2015) was adapted to the circumstances in Italy with the purpose of collecting data concerning the prescription habits of preventive antibiotics among dental practitioners in conjunction with oral implant therapy [17]. The permission of Deeb and co-authors was obtained to use their questionnaire. After being adjusted and translated, the questionnaire was reviewed on comprehensibility and logical order by an experienced Italian oral implantologist. The way the questions were formulated was found appropriate to assess the intended objectives (Additional file 1).

### Setting

Italy is a member state of the European Union, which in 2018 had a population of approximately 60.3 million inhabitants [26]. In March 2018, the number of dentists enrolled with the register held by the National Federation of the Orders of Physicians and Dentists (FNOMCeO) was 61,586 [27].

### Participants

In April 2018, the IAO sent an email to all members of the association (400 dental practitioners) containing a link to a web based questionnaire and a brief introduction regarding the study objectives. All potential respondents received a reminder-email from the IAO after 4 weeks, and 2 weeks later the access to the questionnaire was no longer possible. Furthermore, the participants were guaranteed that the research data would be collected anonymously and the participants had consented the use of the data for the study.

Among all members of the IAO, 36 are female and 20% of all members are actually dentists specialized in oral surgery.

### Variables

Data regarding the following items: demographic details, education, work experience and preventive antibiotic prescribed in case of oral implant placement (including dosage and duration) was gathered. Based on the participants’ answers regarding dosage and period of intake, the total prescribed amount of antibiotics was calculated (mg).

### Data sources / measurement

Each link was directed to a questionnaire that could only be answered once. The questionnaire contained mainly close-ended questions and some open-ended questions.

### Bias

The chance of any bias selection was minimized, since a sample of dentists who are known to regularly carry out oral implants were approached.

### Study size

The final study size included only the dentists, among the ones approached, who had decided to respond partially or completely to the survey.

### Statistical methods

All data was analyzed using STATA® 14 software (StataCorp, College Station, Texas, USA). A statistical evaluation in terms of age, gender and location was carried out. Subsequently, the use of prescribing prophylactic antibiotics and its quantities (mg) before, after or during oral implant surgery was assessed.

The binomial variables corresponding to each of the questions were assessed using proportions (percentage) of the answers to the questionnaire. The chi-squared and Fisher’s exact test were run to evaluate the differences in the antibiotics regimen adopted by the participants according to their gender, age, education, location and work experience.

Eventually, the total dosage of antibiotics in mg being prescribed by each participant was calculated and the mean (mg) was used as the main assessing value. The mean was selected as the main assessing value because of the homogeneity of the sample and its validity and its frequent employment in health research. However, information regarding the median and interquartile range was also provided. ANOVA (Student’s t-test) was run to assess the differences in the total antibiotics (mg) prescribed in concomitance to dental implant surgery. Standard deviation (Std. Dev.) and *P* values were determined in this way.

## Results

### Participants

One hundred and sixty participants returned the survey, resulting in a response rate of 40%.

### Descriptive data

One hundred and forty-six males (93.6%) and ten females (6.4%) answered the questionnaire, who were mostly between 51 and 60 years old (30.1%).

The majority of the participants (97.4%) graduated from a dental school in Italy. Most of the participants graduated from the School of dentistry of Milan (26.9%), others from the School of dentistry of Padova (8.3%) and from the School of dentistry Sapienza - University of Rome (6.4%). Almost two-thirds of the participants (60.9%) had been working as oral health providers for more than 20 years, almost one-third had between 10 and 20 years of experience (30.1%) and the rest of the respondents had been working for less than 10 years (9%). Most of respondents were working in the Lombardia region (30.7%), others in Veneto (10.9%), Lazio (9.6%), Piemonte (7%) and Toscana (7%).

### Outcome data

Approximately 84% of the participants (*n* = 134), currently performing oral implant surgery, stated that they always prescribe prophylactic antibiotics in conjunction with oral implant surgery, only one of the participants (0.6%) never prescribe them.

In addition, 15.6% adopted antibiotics only in particular cases (*n* = 25). Such as cardiopathy requiring antibiotic prophylaxis (24.2%), bone grafting (23.1%); sinus perforation (13.7%); preoperative implant-site infection (11.6%); smokers (9.5%); previous periodontal disease (8.4%); multiple implant insertion (3.1%); medically compromised patients (3.1%) and immediate implant placement (1%). No statistically significant differences were found related to the antibiotic prescriptions of dentists regarding some general characteristics (Table 1).

**Table 1:** Personal characteristics of dentists related to their antibiotic prescription habits in oral implant surgery

Personal characteristics	Antibiotic prescription habits			Total
	*Never*	*Sometimes*	*Always*	
Female ^(a^		8%	6.15%	6.4%
Age (years) ^(b^				
21–30		12%	3.0%	4.5%
31–40		20%	19.2%	19.2%
41–50		24%	30%	28.9%
51–60	100%	24%	30.7%	30.1%
61–70		16%	14.6%	14.7%
71 or more		4%	2.3%	2.6%
Graduation in Italy^(c^	100%	100%	96.1%	96.8%
Experience (years) ^(d^				
Less than 10		16%	7.7%	8.9%
Between 11 and 20	100%	52%	62.3%	60.9%
More than 20		32%	30%	30.1%
Place of settlement (macroregions) ^(e^				
North-West		36%	43.1%	41.7%
North-East	100%	24%	20%	21.1%
Centre		16%	23.1%	21.8%
South		16%	10.8%	11.5%
Islands		4%	2.3%	2.6%
Other		4%	0.7%	1.3%
n^(f^	1	25	130	156

^(a^ *P* = 0.910

^(b^ *P* = 0.735

^(c^ *P* = 0.597

^(d^ *P* = 0.618

^(e^ *P* = 0.718

^(f^ 4 respondents did not or incompletely answer these questions

Most respondents stated that they opt for a combination of a pre- and postoperative regimen (72.9%), while 18.2% only use preoperative regime and 8.8% only post-operative (Table 2).

**Table 2:** Antibiotic prescribing regimens and starting time of the prescriptions

Regimen and prescription starting time	*n*	%	*n*	%
Only pre-operative			29	18.2
Immediately prior	2	6.9		
1 h prior	26	89.6		
1 day prior	0	0		
2 days prior	1	3.4		
Pre- and post-operative^a^			116	72.9
Immediately prior	1	0.8		
1 h prior	59	51.7		
1 day prior	49	42.98		
2 days prior	5	4.39		
Only post-operative			14	8.8
Total			157	100.0

^a^2 respondents did not or incompletely declare their prescriptions starting time

### Main results

#### Pre-operative antibiotics

The majority of the 143 dentists who prescribe preoperative antibiotics when placing oral implants advise their patients to start 1 h prior to surgery (59.4%) or 1 day prior to surgery (34.2%). The other participants prescribing preoperative antibiotics advise starting 2 days (4.2%) or immediately (2.1%) prior to surgery. Table 3 shows the type of antibiotics, its dosage and its regimen.

**Table 3:** Preoperative antibiotic regimens prescribed by dentists

1 h or immediately prior				
*Antibiotic type*	*Dose (mg)*	*Administration*	n	%
Amoxicillin/Clavulanic acid	2.000	oral	32	36.3
Amoxicillin/Clavulanic acid	875/125	oral	22	25
Amoxicillin	2.000	oral	17	19.3
Amoxicillin/Clavulanic acid	1.000	oral	9	10.2
Amoxicillin	1.000	oral	4	4.5
Amoxicillin	500	oral	1	1.1
Penicillin V	1.000	oral	1	1.1
other^a^			2	2.2
Total			88	100.0
**1 or 2 days prior**				
*Antibiotic type*	*Dose (mg)*	*Dosage*	n	%
Amoxicillin/Clavulanic acid	875/125	oral BID	27	49.0
Amoxicillin/Clavulanic acid	1000	oral BID	14	25.4
Amoxicillin	1000	oral BID	8	14.5
Amoxicillin/Clavulanic acid	1000	oral TID	2	3.6
Amoxicillin/Clavulanic acid	800	oral BID	1	1.8
Amoxicillin/Clavulanic acid	875/125	oral TID	1	1.8
Amoxicillin	875/125	oral BID	1	1.8
other^b^	500	oral QD	1	1.8
Total			55	100.0

*QD once a day, BID twice a day, TID 3 times daily, QID 4 times daily*

^a^*1 “Clarithromycin” and 1 “Zithromax PD for 3 days” mentioned spontaneously*

^b^*“Azithromycin 500 mg 1 cpr every 24 h for 3 days” mentioned spontaneously*

^*^2 respondents did not or incompletely declare their prescriptions starting time and their data could not be included in this table

Oral Amoxicillin/Clavulanic acid was found to be the most frequently prescribed antibiotic when administered 1 or 2 days preoperatively (80.7%) and 1 h or immediately prior to surgery (71.6%). Overall, the most frequently preoperative regimen was 2 g of oral Amoxicillin/Clavulanic acid 1 h prior to surgery (*n* = 31, 21.6%).

#### Post-operative antibiotics

Almost three quarters (70.6%) of the dentists who advise patients to start the antibiotics treatment post-operatively, prescribe oral 875/125 mg Amoxicillin/Clavulanic acid twice a day for a period varying from five to six days (Table 4). Overall, The most frequently postoperative regimen prescribed was 875/125 mg oral Amoxicillin/Clavulanic acid twice daily for 6 days after surgery (*n* = 43, 32.5%). Table 4 shows the type of antibiotics, its dosage and its regimen.

**Table 4:** Postoperative antibiotic regimens prescribed by dentists

Antibiotic type	Dose (mg)	Dosage	Duration (days)	n	%
Amoxicillin	1000	oral QD	1	1	0.7
Amoxicillin	1000	oral BID	2	1	0.7
Amoxicillin	1000	oral BID	3	1	0.7
Amoxicillin	1000	oral BID	4	3	2.2
Amoxicillin	1000	oral BID	5	6	4.5
Amoxicillin	1000	oral BID	6	8	6.0
Amoxicillin	1000	oral BID	7	2	1.5
Amoxicillin	500	oral BID	5	1	0.7
Amoxicillin/Clavulanic acid	500/125	oral BID	5	1	0.7
Amoxicillin/Clavulanic acid	500/125	oral TID	6	1	0.7
Amoxicillin/Clavulanic acid	875/125	oral BID	2	3	2.2
Amoxicillin/Clavulanic acid	875/125	oral BID	3	3	2.2
Amoxicillin/Clavulanic acid	875/125	oral BID	4	8	6.0
Amoxicillin/Clavulanic acid	875/125	oral BID	5	28	21.2
Amoxicillin/Clavulanic acid	875/125	oral BID	6	43	32.5
Amoxicillin/Clavulanic acid	875/125	oral BID	7	4	3.0
Amoxicillin/Clavulanic acid	875/125	oral BID	a	1	0.7
Amoxicillin/Clavulanic acid	875/125	oral TID	3	1	0.7
Amoxicillin/Clavulanic acid	875/125	oral TID	4	2	1.5
Amoxicillin/Clavulanic acid	875/125	oral TID	5	3	2.2
Amoxicillin/Clavulanic acid	875/125	oral TID	6	1	0.7
Amoxicillin/Clavulanic acid	875/125	oral TID	7	1	0.7
Penicillin V	875/125	oral BID	7	1	0.7
other^b^				4	3.0
Total				128	100.0

*QD once a day, BID twice a day, TID 3 times daily, QID 4 times daily*

^a^*not responded*

^b^*1 “Azithromycin 500 mg”, 1“Clarithromycin”, 1 “Clarithromycin ×2 250 mg ×5 a day* per os*” and 1 “Zithromax PD for 3 days”*

#### Amount of prescribed antibiotics

On average, dentists prescribed a total of 10,331 mg of antibiotics (Standard deviation = 4973 mg) before, after or during oral implant placement, varying form 1000 mg to 22,000 mg. Dentists who prescribed only preoperative antibiotics administered, on average, significantly (*p* = 0.000) less mg (2241 mg) than their colleagues who prescribed antibiotics only after surgery (10,404 mg), or prior and after surgery (12,436 mg).

No statistically significant differences (*p* = 0.176) were found in the mean values of the total amount of antibiotics prescribed (mg) by dentists who routinely prescribed prophylactic antibiotics compared to those who prescribed antibiotics not on a regular basis.

#### Antibiotic regimens in case of penicillin allergies

**Fig. 1:**
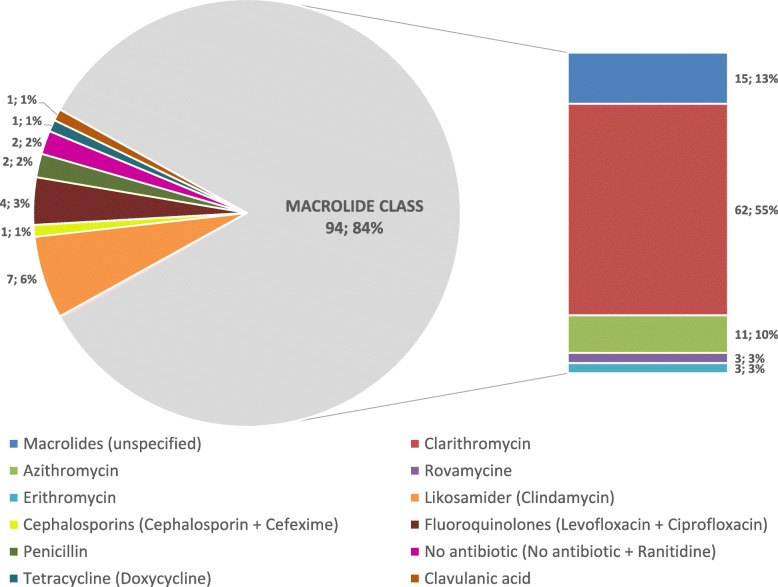
Antibiotics prescribed to patients allergic to penicillin

Participants prescribed a large range of different prophylactic antibiotics to patients allergic to penicillin. Overall, 12 different antibiotics types were prescribed; 94 participants prescribed macrolides, and one participant prescribed none at all. The majority of participants (*n* = 62, 52.9%) prescribed Clarithromycin instead (Fig. 1).

#### Compliance with last published evidence

Approximately, only 17% (*n* = 27) of the participants who prescribed antibiotics before oral implant surgery adhered to recommendations proposed by the latest publications (no more than 3 g of preoperative amoxicillin before oral implant surgery) [2, 7]. Of these, 25 began prescribing antibiotics 1 h before the intervention prescribing Amoxicillin (*n* = 11) or Amoxicillin/Clavulanic (*n* = 14). Prescriptions made immediately before the intervention always contained Amoxicillin/Clavulanic. Overall, the most commonly prescribed regimen among these participants was 2 g of oral Amoxicillin 1 h before surgery (*n* = 10).

## Discussion

### Key results

Bearing in mind the last published evidence on this topic, most of the dentists surveyed in this study did not comply with their recommendations [2, 7]. They systematically prescribed antibiotics in oral implant surgery to healthy patients, frequently using extended postoperative treatments. In addition, there is currently in Italy a discordance about the antibiotic type and regimen selected, especially when treating patients allergic to penicillin.

### Limitations

It is unknown as such the number of dentists placing implants in Italy. Therefore, this sample is based on dentists acknowledged as dental practitioners performing oral implant surgery in Italy. The large differences shown in the gender of participants may be related to the low rate of IAO female members. The response rate of 40% was quite low but it was retained satisfactory for a web survey [28]. Nevertheless, this fact could be a potential risk of bias because it is unknown whether the drop-outs are over-prescribing professionals or they are just uninterested in this topic.

Despite being uncertain whether all dentists placing oral implants in Italy were reached, the study sample could not be considered unrepresentative of the target population (members of the IAO).

The survey was completely anonymous to protect the participants’ privacy as well as to insure sincere answers. However, truthful answers are not always possible. As in most cross-sectional surveys, what participants declare about their therapies is not always in accordance with their authentic treatment.

In order to favor comparability, this survey was based on a questionnaire performed in the USA. A specialized translation company translated that questionnaire from English to Italian but it was not translated back to English to check correct phrasing order. Instead, an experienced Italian implantologist checked its comprehensively and logical order. Before translation, the original questionnaire was adjusted to circumstances in Italy but it was not validated.

### Interpretation

The large range of different regimens prescribed by dentists in this study confirmed that there is not a standard prophylactic antibiotic regimen prescribed to healthy patients undergoing oral implant surgery in Italy. This has already been shown in other medical procedures where the antibiotic prophylaxis is elective according to each physician [29]. This is also being the case among oral health professionals in other countries [13–18].

Despite having no guidelines available in Italy validating the regular prescription of prophylactic antibiotics in oral implant surgery to healthy patients, this study has shed light on the fact that the majority of dentists in Italy are routinely prescribing long antibiotic treatments to healthy patients without a substantial indication.

Regarding the most recent reviews published on this topic, prophylactic antibiotics have not been found beneficial in preventing postoperative infections. Just a single preoperative dose of amoxicillin (1 g, 2 g or 3 g) prior to oral implant placement might prevent oral implant failure among healthy patients [2, 7]. Consequently, the prescription of postoperative antibiotics in healthy patients could be considered overtreatment and it could lead to potential adverse reactions and unnecessary costs.

Unfortunately, most of dentists surveyed in this study commonly prescribed longer regimens including postoperative antibiotics instead. Most participants consistently prescribed various types of antibiotics and prophylactic regimens without any scientific-based support. The absence of standardized guidelines could be considered an important reason for the discretional use of antibiotics. Moreover, a lack of scientific evidence on the use of further antibiotic types (different to amoxicillin) might be the reason of such large variation when treating patients allergic to penicillin.

A similar survey performed among 109 dentists in UK found that approximately 72% of dentists prescribed antibiotics for all oral implant surgeries [14]. Other analogous study performed among 133 dentists in Sweden showed nearly the same data (74%) [15]. This percentage was considerably lower among 176 dentists in Jordan (50%) [13]. On the other hand, the percentage prescribing prophylactic antibiotics for healthy patients among 217 maxillofacial surgeons in the USA (96%) and among 233 dentists in Spain (90%) was slightly higher than in Italy [17, 18].

In Italy, significant differences in the means of prescribed antibiotics (mg) were found between dentists prescribing only preoperative antibiotics and those prescribing only postoperative or pre- and postoperative regimens. This may be due to the dispersion of the variables (difference in variances), or a real statistically significant difference in their means. The analysis of variance did not offer an explanation but the durations of exclusive preoperative regimens were frequently shorter and this might be a plausible explanation.

The current condition described on this cross-sectional survey may produce a negative discrepancy in the risk-benefit ratio concerning the use of prophylactic antibiotics because of a reduction of their positive effects and an increasing incidence of adverse reactions such as bacterial resistance, patient risk and societal costs.

### Generalizability

This cross-sectional survey was performed among dentists acknowledge in Italy as professionals who carry out oral implant surgery and who have graduated in representative proportions from different Italian dental schools. The survey has an internal validity (lack of bias in estimating the dentist’s current antibiotic prescribing habits in combination with oral implant surgery) for its target population (members of the IAO). Furthermore, this study has also an external validity (lack of bias to extrapolate its estimations) for an external population (all dentists placing oral implants in Italy). The authors found no epidemiological reason indicating that our target population differs from all dentists’ population currently placing oral implants in Italy. Therefore, the authors assumed that the estimates from this survey could be extrapolated to all dentists currently performing oral implant surgery in Italy.

## Conclusions

Dentists in Italy on a large scale prescribe antibiotic prophylaxis in conjunction with oral implant surgery among healthy patients. A high range of prophylactic regimens is prescribed and they are not adhering to the science-based recommendations [2, 7]. Guidelines based on last published evidence and focused on the indications for prophylactic antibiotics among healthy patients (also for those allergic to penicillin) are required to prevent bacterial resistance, side effects and costs caused by overtreatment and the irrational use of antibiotics.

## Supplementary information

**Additional file 1.** Questionnaire. This file contains the English version of the survey sent to IAO members.

## Data Availability

The datasets used and/or analyzed during the current study are available from the corresponding author on reasonable request.

## Abbreviations

EU: European Union
FNOMCeO: Orders of Physicians and Dentists
g: Grams
IAO: Italian Academy of Osseointegration
mg: Milligrams
SICOI: Italian Society of Oral Surgery and Implant Dentistry
SIO: Italian Society of Osseointegration
Std. Dev: Standard deviation
STROBE: Strengthening the Reporting of Observational studies in Epidemiology
UK: United Kingdom
USA: United States of America
