# Supplementary material for: Antibiotic prophylaxis habits in oral implant surgery among dentists in Italy: a cross-sectional survey
Source: BMC Oral Health. 2019 Dec 2;19:265. doi: 10.1186/s12903-019-0943-x (PMC6889412; doi:10.1186/s12903-019-0943-x)
Supplement: Supplementary file 1 — Additional file 1. Questionnaire. This file contains the English version of the survey sent to IAO members. [file 12903_2019_943_MOESM1_ESM.pdf]

Survey: I- Antibiotic prophylaxis habits in oral implant surgery.

Page 1.- Beginning of the Survey.

Please answer the following question:

Do you prescribe antibiotics before, during or after the insertion of dental implants?

(* Required question).
(* Mark only one option)

☐ No, I haven't.
☐ Yeah, sometimes.
☐ Yeah, all the time.

If your answer is "Yes, sometimes" please indicate under which circumstances you prescribe antibiotics:

(* Required question).

(*Answer only if:
The answer to "Do you prescribe antibiotics before, during, or after inserting dental implants?" **is**: "Yes sometimes" on the "Start Survey" page. .)

☐ 1. Bone insert surgery
☐ 2. Patient with previous peridontal pathologies
☐ 3. Patient smoker
☐ 4. Pre-operative implant site infection
☐ 5. Perforation of the maxillary sinus
☐ 6. Simultaneous insertion of more than one dental implant
☐ 7. Cardiopathy requiring antibiotic prophylaxis
☐ 8. Other circumstances (Specify which)____

Jump logic.

Time model for antibiotic prescription, only if:
the answer to "Do you prescribe antibiotics before, during or after the insertion of dental implants? **"is**: "Yes, sometimes" on the "Start Survey" page.
or the answer to "Do you prescribe antibiotics before, during, or after inserting dental implants?" **is**: "Yes, always" on the "Start Survey" page.
Go to: "Page 5.- Demographic data and Conclusion of the Survey", only if:
the answer to "Do you prescribe antibiotics before, during, or after the insertion of dental implants?" **is**: "No, never" on the "Start Survey" page.

Time model of antibiotic prescription

We invite you to answer the following questions on the assumption that your patients are healthy and have no allergy to antibiotics. Choose the option that is most in tune with or closest to your clinical practice.

Dom.2. - At what time does it prescribe Antibiotics?

(*Question to be answered obligatorily)
(* Mark only one option)

☐ Exclusively before surgery (Pre-operators)
☐ Only after surgery (Post-operative)
☐ Before and after surgery (Pre and Post-operative)

Jump logic.

Go to: "Page 2.- Pre-operative antibiotic prescription model (before insertion of the dental implant)", only if:
the answer to "At what time do you prescribe Antibiotics?" **is**: "Only before surgery (Pre-operators)" on the page "Antibiotic prescription time model".
Go to: "Page 4.- Post-operative antibiotic prescription model (after insertion of the dental implant)", only if:
the answer to "At what time do you prescribe Antibiotics?" **is**: "Only after surgery (Post-operatives)" on the page "Time

model of antibiotic prescription".

Model of pre-operative antibiotic prescription (before insertion of the dental implant).

We invite you to answer the following questions on the assumption that your patients are healthy and have no allergy to antibiotics. Choose the option that is most in tune with or closest to your clinical practice.

When does the prophylaxis antibiotic start to be applied before the implants are inserted?

(* Required question).
(* Mark only one option)

- ☐ 2 days before surgery.
- ☐ 1 day before surgery.
- ☐ 1 hour before surgery.
- ☐ Immediately before surgery.

You have selected "1 or 2 days before", please indicate only one type of antibiotic among those listed below:

(* Required question).
(* Mark only one option)

(*Answer only if:
the answer to "When does the prophylaxis antibiotic start to be applied before the implants are inserted" **is**: "2 days before the surgery" on the page "Pre-operative antibiotic prescription model (before the insertion of the dental implant)". or
the answer to "When does the prophylaxis antibiotic start to be applied before the implants are inserted" **is**: "1 day before the surgery" on the page "Pre-operative antibiotic prescription model (before the insertion of the dental implant)".)

- ☐ Amoxicillin.
- ☐ Amoxicillin/Clavulanic.
- ☐ Clindamycin.
- ☐ Penicillin V.
- ☐ Cefalexin.
- ☐ Erythromycin.
- ☐ Other antibiotics.

You have selected "1 hour before" or "Immediately before", please indicate only one type of antibiotic among those listed below:

(* Required question).
(* Mark only one option)

(*Answer only if:
the answer to "When does the antibiotic prophylaxis start to be applied before the implants are inserted" **is**: "1 hour before the surgery" on the page "Pre-operative antibiotic prescription model (before the dental implant is inserted)" or "1 hour before the implants are inserted" on the page "Pre-operative antibiotic prescription model (before the dental implant is inserted)"?
the answer to "When does the prophylaxis antibiotic start to be applied immediately before implant placement?" **is**: "Immediately before surgery" on the page "Pre-operative antibiotic prescription model (before implant placement)". .)

- ☐ Amoxicillin.
- ☐ Amoxicillin/Clavulanic.
- ☐ Clindamycin.
- ☐ Penicillin V.
- ☐ Cefalexin.
- ☐ Erythromycin.
- ☐ Ampicillin
- ☐ Cefazolin
- ☐ Other antibiotics.

Please specify the dosage, the dosage and the route of administration (1 or 2 days before):

(* Required question).
(* Mark only one option per group)

(*Answer only if:
the answer to "You selected "1 or 2 days before", please indicate only one type of antibiotic among those listed below:" **is:** Amoxicillin." on the page "Pre-operative antibiotic prescription model (before insertion of the dental implant)." or
the answer to "You selected "1 or 2 days before", please indicate only one type of antibiotic among those listed below:" **is:** Amoxicillin/Clavulanic." on the page "Pre-operative antibiotic prescription model (before insertion of the dental implant)" or
the answer to "You selected "1 or 2 days before", please indicate only one type of antibiotic among those listed below:" **is:** "Clindamycin." on the page "Pre-operative antibiotic prescription model (before insertion of the dental implant)." or
the answer to "You selected "1 or 2 days before", please indicate only one type of antibiotic among those listed below:" **is:** Penicillin V." on the page "Pre-operative antibiotic prescription model (before insertion of the dental implant)" or
the answer to "You selected "1 or 2 days before", please indicate only one type of antibiotic among those listed below:" **is:** Cefalexin." on the page "Pre-operative antibiotic prescription model (before insertion of the dental implant)." or
the answer to "You selected "1 or 2 days before", please indicate only one type of antibiotic among those listed below:" **is:** "Erythromycin." on the "Pre-operative antibiotic prescription model page (before insertion of the dental implant)" page.
.)

Dosage (mg)	Dosage	Route of administration
☐ 150	☐ 1 time a day.	☐ Oral.
☐ 250	☐ Twice a day.	☐ Intramuscular.
☐ 300	☐ Three times a day.	☐ Endovenous.
☐ 400	☐ Four times a day.	
☐ 500		
☐ 500/125		
☐ 800		
☐ 875/125		
☐ 1000		

Please specify the dosage, dosage and route of administration (1 hour or immediately before):

(* Required question).
(* Mark only one option per group)

(*Answer only if:
the answer to "You have selected "1 hour before" or "Immediately before", please indicate only one type of antibiotic among those listed below:" **is**: "Amoxicillin." on the page "Pre-operative antibiotic prescription model (before insertion of the dental implant)". or
the answer to "You have selected "1 hour before" or "Immediately before", please indicate only one type of antibiotic among those listed below:" **is**: "Amoxicillin/Clavulanic." on the page "Pre-operative antibiotic prescription model (before insertion of the dental implant)" or "Dental implant prescription model".
the answer to "You have selected "1 hour before" or "Immediately before", please indicate only one type of antibiotic among those listed below:" **is**: "Clindamycin." on the page "Pre-operative antibiotic prescription model (before insertion of the dental implant)". or
the answer to "You have selected "1 hour before" or "Immediately before", please indicate only one type of antibiotic among those listed below:" **is**: "Penicillin V." on the page "Pre-operative antibiotic prescription model (before insertion of the dental implant)". or
the answer to "You have selected "1 hour before" or "Immediately before", please indicate only one type of antibiotic among those listed below:" **is**: "Cefalexin." on the page "Pre-operative antibiotic prescription model (before insertion of the dental implant)". or
the answer to "You have selected "1 hour before" or "Immediately before", please indicate only one type of antibiotic among those listed below:" **is**: "Erythromycin." on the page "Pre-operative antibiotic prescription model (before insertion of the dental implant)". or
the answer to "You have selected "1 hour before" or "Immediately before", please indicate only one type of antibiotic among those listed below:" **is**: "Ampicillin." on the page "Pre-operative antibiotic prescription model (before insertion of the dental implant)". or
the answer to "You have selected "1 hour before" or "Immediately before", please indicate only one type of antibiotic among those listed below:" **is**: "Cefazolin" on the page "Pre-operative antibiotic prescription model (before insertion of the dental implant)". .)

Dosage (mg)	Dosage	Route of administration
☐ 500	☐ One time only.	☐ Oral.
☐ 500/125		☐ Intramuscular.
☐ 600		☐ Endovenous.
☐ 800		

☐ 875/125
☐ 1000
☐ 1600
☐ 2000

If you have selected "Other antibiotics", please specify the name, dosage, dosage and route of administration used:

(* Required question).

(*Answer only if:
the answer to "You selected "1 or 2 days before", please indicate only one type of antibiotic among those listed below:" **is:** Other antibiotics" on the page "Pre-operative antibiotic prescription model (before insertion of the dental implant)" or
the answer to "You have selected "1 hour before" or "Immediately before", please indicate only one type of antibiotic among those listed below:" **is**: "Other antibiotics." on the page "Pre-operative antibiotic prescription model (before insertion of the dental implant)". .)

____

Jump logic.

Go to: "Page 5.- Demographic data and Conclusion of the Survey", only if:
the answer to "At what time does he prescribe antibiotics? **"is**: "Only before surgery (Pre-op)" on the page "Antibiotic prescription time model".

Model of post-operative antibiotic prescription (after insertion of the dental implant).

We invite you to answer the following questions on the assumption that your patients are healthy and have no allergy to antibiotics. Choose the option that is most in tune with or closest to your clinical practice.

Select the antibiotic it prescribes after the insertion of the dental implants (It is POSSIBLE to CHOOSE ONE TYPE OF ANTIBIOTICAL):

(* Required question).
(* Mark only one option)

☐ Amoxicillin.
☐ Amoxicillin/Clavulanic.
☐ Clindamycin.
☐ Penicillin V.
☐ Cefalexin.
☐ Erythromycin.
☐ Other antibiotics.

Please specify the dosage, the dosage, the route of administration and the duration of the antibiotic treatment:

(* Required question).
(* Mark only one option per group)

(*Answer only if:
the answer to "Select antibiotic prescribed after insertion of dental implants (ONE TYPE OF ANTIBIOTICAL CHOICE IS POSSIBLE):" **is**: "Amoxicillin." on the page "Post-operative antibiotic prescribed model (after insertion of dental implants)." or "Patient's antibiotic prescribed model".
the answer to "Select the antibiotic that you prescribe after insertion of dental implants (you can choose one single type of antibiotic):" **is**: "Amoxicillin/Clavulanic." on the page "Model of post-operative antibiotic prescription (after insertion of dental implants)." or
the answer to "Select antibiotic prescribed after insertion of dental implants (ONE TYPE OF ANTIBIOTICAL CHOICE IS POSSIBLE):" **is**: "Clindamycin." on the page "Post-operative antibiotic prescribed model (after insertion of dental implants)" or "Dental implant prescribed model".
the answer to "Select antibiotic prescribed after insertion of dental implants (ONE TYPE OF ANTIBIOTICAL CHOICE IS POSSIBLE):" **is**: "Penicillin V." on the page "Post-operative antibiotic prescribed model (after insertion of dental implants)". or
the answer to "Select antibiotic prescribed after insertion of dental implants (ONE TYPE OF ANTIBIOTICAL CHOICE IS POSSIBLE):" **is**: "Cefalexin." on the page "Post-operative antibiotic prescribed model (after insertion of dental implants)" or

Universidad del País Vasco / Euskal Herriko Unibertsitatea — ITALIAN ACADEMY OF OSSEOINTEGRATION

"Dental implant prescribed model".
the answer to "Select the antibiotic that you prescribe after insertion of dental implants (you can choose one single type of antibiotic):" **is**: "Erythromycin." on the page "Model of post-operative antibiotic prescription (after insertion of dental implants)". .)

Dosage (mg)	Dosage	Route of administration	Duration of treatment (days)
☐ 150	☐ 1 time a day.	☐ Oral.	☐ 1
☐ 250	☐ Twice a day.	☐ Intramuscular.	☐ 2
☐ 300	☐ Three times a day.	☐ Endovenous.	☐ 3
☐ 400	☐ Four times a day.		☐ 4
☐ 500			☐ 5
☐ 500/125			☐ 6
☐ 800			☐ 7
☐ 875/125			☐ 8
☐ 1000			☐ 9
			☐ 10
			☐ 11
			☐ 12
			☐ 13
			☐ 14
			☐ 15

You have selected "Other antibiotics", specify the name, dosage, dosage and route of administration used:

(* Required question).

(*Answer only if:
the answer to "Select the antibiotic that you prescribe after insertion of dental implants (you can choose one single type of antibiotic):" **is**: "Other antibiotics." on the page "Model of post-operative antibiotic prescription (after insertion of dental implant)". .)

____

Model of antibiotic prescription for patients allergic to penicillins.

We invite you to answer the following questions based on the assumption that your patients are healthy but have allergies to penicillins and their derivatives.

Describe the antibiotic (active substance) and the dosage model you would prescribe if your patient was allergic to penicillins:

(* Required question).

____

Demographic data and Conclusion of the Survey.

We would like to thank you for your cooperation and time spent filling out our anonymous questionnaire.
Finally, we ask you to complete the survey with the demographic data indicated below. Thank you. [Chuckles]

Sun.6.- Genre:

(* Required question).
(* Mark only one option)

☐ Female
☐ Male

Sun.7.- Age group:

(* Required question).
(* Mark only one option per group)

-

Age (years)

☐ 21-30
☐ 31-40
☐ 41-50
☐ 51-60
☐ 61-70
☐ over 70 years

Dom.8.- University attended:

(* Required question).
(* Mark only one option)

You answered "Other Universities" to the previous question: please specify the name of the university you attended and the country in which you obtained your degree:

(*Answer only if:
The answer to "University attended:" **is**: "Other universities" on the page "Demographic data and Conclusion of the Survey." .)

____

Choose from the answers listed below the one that best corresponds to your years of clinical experience in patient care:

(* Required question).

☐ Less than 10 years old.
☐ Between the ages of 11 and 20.
☐ Over 20 years

Dom.10.- Please indicate the Postal Code of the facility (hospital, clinic or practice) where you practice your oral implantology activity.

(* Required question).

____
